# Insidious chromatin change with a propensity to exhaust intestinal stem cells during aging

**DOI:** 10.1016/j.isci.2024.110793

**Published:** 2024-09-09

**Authors:** Saki Tomita-Naito, Shivakshi Sulekh, Sa Kan Yoo

**Affiliations:** 1 Laboratory for Homeodynamics, RIKEN BDR, Kobe, Japan; 2 Graduate School of Frontier Biosciences, Osaka University, Osaka, Japan; 3 Division of Developmental Biology and Regenerative Medicine, Kobe University, Kobe, Japan; 4 Physiological Genetics Laboratory, RIKEN CPR, Kobe, Japan

**Keywords:** Molecular biology, Cell biology, Omics, Transcriptomics

## Abstract

During aging, tissue stem cells can demonstrate two opposing phenotypes of tissue homeostasis disruption: proliferation and exhaustion. Stem cells can exhaust as a result of excessive cell proliferation or independently of cell proliferation. There are many silent changes in chromatin structures and gene expression that are not necessarily reflected in manifested phenotypes during aging. Here through analyses of chromatin accessibility and gene expression in intestinal progenitor cells during aging, we discovered changes of chromatin accessibility and gene expression that have a propensity to exhaust intestinal stem cells (ISCs). During aging, Trithorax-like (Trl) target genes, *ced-6* and *ci*, close their chromatin structures and decrease their expression in intestinal progenitor cells. Inhibition of *Trl*, *ced-6*, or *ci* exhausts ISCs. This study provides new insight into changes of chromatin accessibility and gene expression that have a potential to exhaust ISCs during aging.

## Highlights

• Chromatin and gene expression change in intestinal stem cells (ISCs) during aging
• Some changes of chromatin and gene expression have a propensity to exhaust ISCs
• Trithorax-like target genes close their chromatin and decrease expression during aging
• Inhibition of Trithorax-like or its target genes exhausts ISCs during aging

## Graphical abstract

**Figure:**
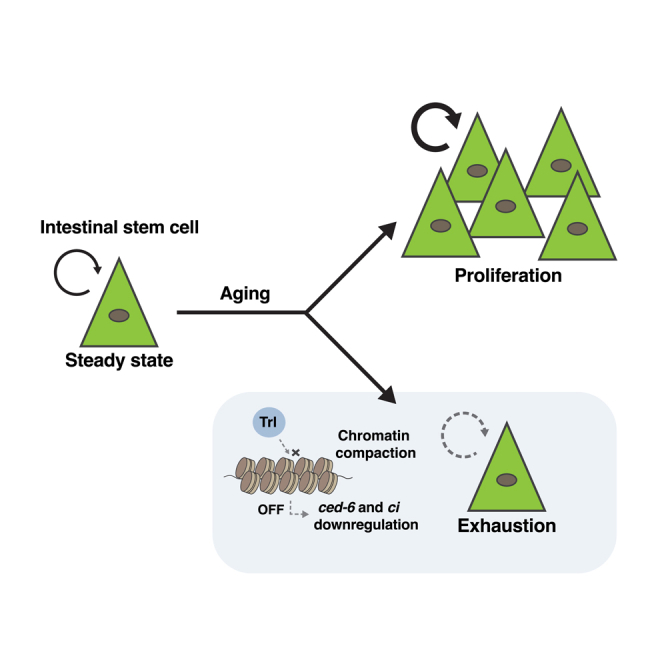

## Introduction

During aging, miscellaneous changes occur in tissue stem cells. Tissue stem cells often exhibit two opposite phenotypes: proliferation and exhaustion.^1^^,^^2^ Proliferation can lead to dysplasia and tumorigenesis. Stem cell exhaustion is often defined as a decline in stem cell numbers and renewal capacity.^1^^,^^2^^,^^3^ Although stem cell quiescence and exhaustion share the same property of suppressed proliferation, they are distinct in a sense that quiescent cells, but not exhausted cells, can proliferate upon receiving stresses. Thus, stem cell exhaustion can be defined as a stress-induced cellular status exhibiting decrease of either the cell number or proliferative capacity, which makes stem cells refractory to stimulation and unable to renew upon receiving additional stresses.

Aging-induced stem cell exhaustion occurs in many types of tissue stem cells in mice, including hematopoietic stem cells, intestinal stem cells (ISCs), skeletal muscle stem cells, and hair follicle stem cells.^3^^,^^4^^,^^5^^,^^6^^,^^7^^,^^8^ Stem cell exhaustion can occur due to two mechanisms: (1) replicative stress in response to proliferation and (2) mechanisms independent of cell proliferation.^2^^,^^3^^,^^4^^,^^5^^,^^6^^,^^7^^,^^8^^,^^9^ The resulting phenotype, proliferation or exhaustion, likely depends on the tug of war competition between conflicting signals.

In *Drosophila*, ISCs demonstrate a proliferative phenotype during aging.^10^^,^^11^ Many studies focused on what is driving aging-induced ISC proliferation and elucidated the mechanisms such as JNK signaling,^10^ commensal dysbiosis,^12^ epithelial barrier disruption,^13^ mitochondrial regulation,^14^^,^^15^ and an ABC transporter-mediated folate accumulation.^16^ Although PIWI was suggested to suppress Jak-Stat-mediated exhaustion of ISCs,^9^ signaling that skews ISCs toward exhaustion during aging is not known. There might be some undiscovered signals that lead cells toward exhaustion.

During aging, changes in chromatin structures and gene expression occur simultaneously in tissue stem cells.^17^^,^^18^ Changes in chromatin structures may underlie changes of some gene expression. How these alterations during aging affect the cellular phenotype remains to be elucidated. Here we addressed a potential mechanism of ISC exhaustion during aging by focusing on chromatin accessibility and gene expression.

## Results

We previously showed that inhibition of White, an ABC transporter, suppresses aging-induced ISC proliferation through folate metabolism suppression.^16^ We noticed that, when we inhibited ISC proliferation by the *white* mutation, ISC proliferation is rather decreased at the old age (Figure S1). The literature also suggests a similar phenomenon: when JNK, which induces ISC proliferation, is inhibited, ISC proliferation is reduced at old ages compared to younger ages.^19^ This led to germination of an idea that there might be some proliferation-independent, silent signaling with a potential to exhaust ISCs during aging, which is usually masked or competed out by more dominant proliferating signaling.

**Figure 1:**
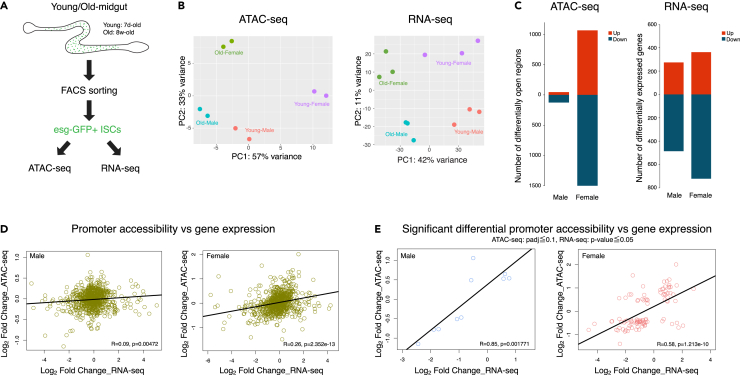
ATAC-seq and RNA-seq of intestinal progenitor cells during aging (A) Experimental flow of intestinal progenitor cell isolation for ATAC-seq and RNA-seq. GFP-expressing progenitor cells were isolated from young and old guts by FACS. (B) Principal-component analysis (PCA) of chromatin openness (left) and transcription(right) in young and old intestinal progenitor cells from both sexes. (C) Differential accessibility/expression analysis in male and female intestinal progenitor cells (left, ATAC- seq; right, RNA-seq). Bar plots indicate the number of upregulated regions/genes (red) and the number of downregulated regions/genes (blue) during aging. Minimum fold change = 2, adjusted *p* value < 0.1. Statistical significance was determined using the Benjamini-Hochberg procedure and two-tailed unpaired t test. (D) Correlation plots between promoter accessibility and gene expression in males and females. R value indicates Pearson’s product-moment correlation coefficient. (E) Correlation plots between significantly differential promoter accessibility and gene expression (ATAC-seq; padj <= 0.1, RNA-seq; *p* value <= 0.05). Statistical significance was determined using the Benjamini-Hochberg procedure and two-tailed unpaired t test.

We decided to obtain descriptive data to investigate potential exhaustion signaling. Since stem cell exhaustion in mammals often involves epigenetic remodeling of chromatin,^20^^,^^21^ we focused on chromatin dynamics during aging. We performed ATAC-seq and RNA sequencing (RNA-seq) using isolated intestinal progenitor cells to elucidate molecular changes that are masked or competed out by the proliferation phenotype. We isolated the intestinal progenitor cells in both male and female progeny from a cross of OregonR and *esg-GFP* (P01986) at young and old ages by fluorescence-activated cell sorting (FACS) (Figure 1A). GFP-positive cells include ISCs, enteroblasts, and misdifferentiated esg-GFP-positive cells. Principal-component analysis indicates that, in both sexes, aging affects gene expression and chromatin accessibility (Figure 1B). Notably, although there are changes in chromatin accessibility in males during aging, its magnitude is much less pronounced compared to females (Figures 1B and 1C). Gene expression changed to a similar degree in both sexes (Figures 1B and 1C). Both directions of changes, opening and closing in chromatin and up- and downregulation in transcription, were observed during aging, albeit slightly more dominant toward closing and downregulation. Overall, there was very little correlation between chromatin accessibility and gene expression (Figure 1D). When we focused on genes that have significant changes in both ATAC-seq and RNA-seq during aging, there was a correlation (Figure 1E). These findings are consistent with the previous analyses performed with female ISCs.^22^ This indicates that, although changes of chromatin accessibility and gene expression occur independently at a global level, at local levels some gene expressions might be affected by chromatin structural changes.

**Figure 2:**
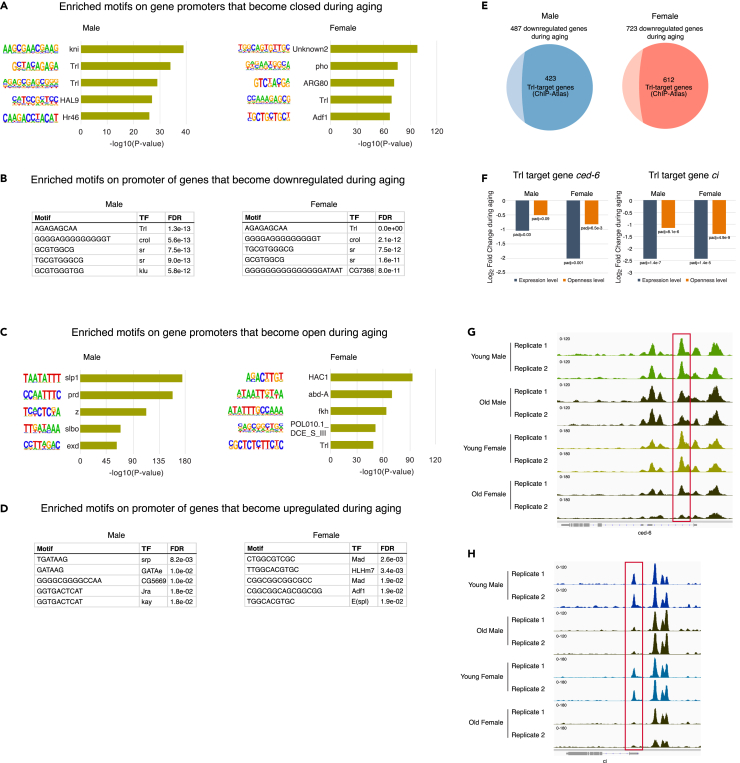
Decrease in chromatin accessibility and transcription of Trl target genes during aging (A) TF (transcription factor) motif enrichment analysis on promoter peaks that become closed during aging. Young peak data were compared to old peak data. (B) TF motif enrichment analysis on promoters of genes that become downregulated during aging. (C) TF motif enrichment analysis on promoter peaks that become open during aging. Old peak data were compared to young peak data. (D) TF motif enrichment analysis on promoters of genes that become upregulated during aging. (E) Venn diagram showing the overlap between downregulated genes during aging and Trl target genes from ChIP-Atlas database. (F) Expression levels and chromatin openness levels of Trl target gene *ced-6* and *ci* decrease during aging. Statistical significance was determined using the Benjamini-Hochberg procedure. (G and H) Chromatin accessibility in the region of ced-6 and ci in two replicates. The red box indicates promoter accessibility showing a significant difference during aging.

A motif enrichment analysis with the ATAC-seq data demonstrates that, during aging, genes regulated by the chromatin regulator Trithorax-like (Trl) tend to change chromatin accessibility toward being both closed and open in females and being closed in males (Figures 2A and 2C). This observation overlaps with the previous report that Trl binding sites are enriched in promoter regions that become open during aging in female ISCs^22^ but is distinct in that closing chromatin also enriches Trl binding sites in both sexes. We also found, through a motif analysis with the RNA-seq data by iDEP,^23^ that Trl target genes tend to be downregulated in both sexes (Figures 2B–2D). Based on the ChIP-Atlas,^24^ we confirmed that many genes downregulated in old ISCs are Trl target genes (423 of 487 genes in females and 612 of 723 genes in males) (Figure 2E). Since we observed chromatin closing and transcription reduction of the Trl target genes during aging regardless of sex, we considered that these changes should be the most general change without sexual dimorphism. It is of note that *Trl* expression itself did not change during aging (Figure S2A), indicating that the changes of Trl target gene expression are likely mediated by chromatin structural changes.

*Trl* encodes GAGA-associated factor that regulates enhancer-promoter and promoter-promoter interactions, gene transcription, and chromatin accessibility.^25^^,^^26^^,^^27^^,^^28^ *Trl* mutants exhibit a cell proliferation defect in embryos,^25^^,^^26^ suggesting that genes regulated by Trl could mediate potential exhaustion signals. Among Trl target genes, we focused on *ced-6* and *cubitus interruptus (ci)*, both of which simultaneously exhibit closing of chromatin accessibility and downregulation of transcription in aging intestinal progenitor cells (Figures 2F–2H). Ced-6 is known to regulate cellular engulfment together with Draper (Drpr).^29^^,^^30^^,^^31^ Ci, a transcription factor downstream of hedgehog signaling, has previously been shown to regulate ISC proliferation.^32^

**Figure 3:**
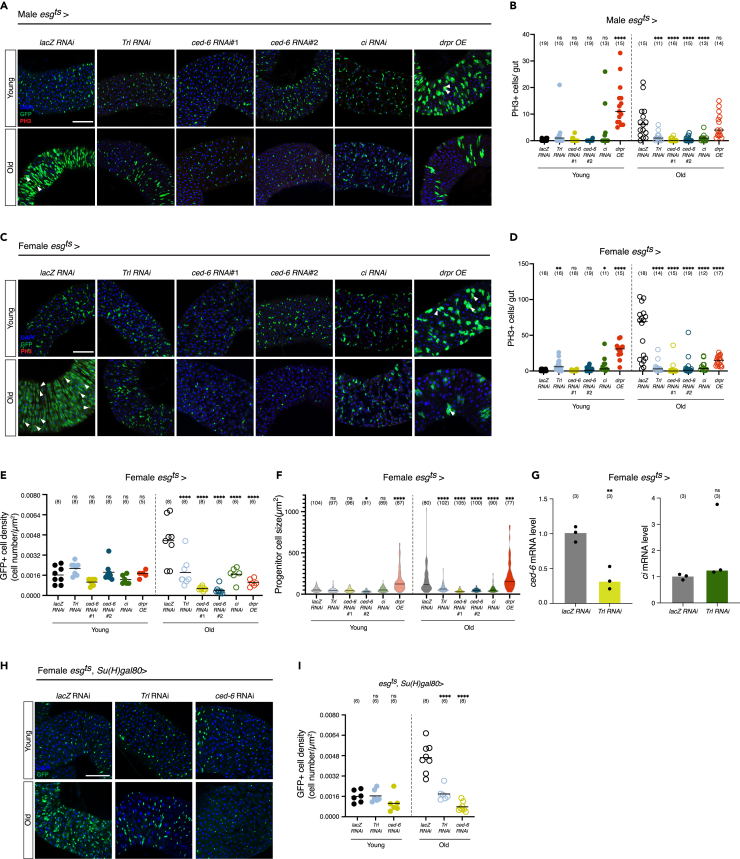
Inhibition of Trl and its target genes induce ISC exhaustion during aging (A and C) Knockdown of Trl or its target gene ced-6 or ci suppresses aging-induced ISC proliferation. Confocal images show posterior midguts (R4) from male (A) and female (C) flies expressing RNAi against lacZ, Trl, ced-6, and ci, or overexpressing drpr. esg-driven GFP expression (green, marking progenitors), phospho-histone 3 (PH3) immunostaining (red), and DAPI (blue) are shown in the images. Scale bars, 100 μm. Arrowheads point to PH3+ cells. (B and D) Quantification of PH3+ cells per midgut in male (B) and female (D) flies. ∗*p* < 0.05, ∗∗*p* = 0.0083, ∗∗∗*p* < 0.001, ∗∗∗∗*p* < 0.0001; one-way ANOVA with Dunnett’s multiple comparisons test. *n* = 11–19 flies. Black lines indicate median. (E) Quantification of GFP+ cell density. ∗∗∗∗*p* < 0.0001; one-way ANOVA with Dunnett’s multiple comparisons test. *n* = 5–8 flies. Black lines indicate mean. (F) Quantification of progenitor cell size (μm^2^). ∗*p* < 0.05, ∗∗∗*p* < 0.001, ∗∗∗∗*p* < 0.0001; one-way ANOVA with Dunnett’s multiple comparisons test. *n* = 77–105 GFP+ cells. 8 midguts (R4) were analyzed per sample. Black lines indicate median. (G) RT-qPCR of ced-6 and ci mRNA in progenitor cells isolated from female Trl knockdown flies. ∗∗*p* = 0.0047; two-tailed unpaired t test. *n* = 3 replicates. Bars indicate median. (H) Confocal images show posterior midguts isolated from female flies expressing RNAi against lacZ, Trl, and ced-6 driven by esg-gal4 with Su(H)-gal80. GFP (green, marking ISCs) and DAPI (blue) are shown in the images. Scale bars, 100 μm. (I) Quantification of GFP+ cell density in (H). ∗∗∗∗*p* < 0.0001; one-way ANOVA with Dunnett’s multiple comparisons test. *n* = 6–8 flies. Black lines indicate mean.

We inhibited *Trl, ced-6*, or *ci* in intestinal progenitor cells of young and old animals with RNAis that are either previously validated^33^^,^^34^^,^^35^ or validated by ourselves (Figure S2B). Knockdown of *Trl*, *ced-6*, or *ci* suppressed aging-induced ISC proliferation, which was detected by the mitotic marker phospho-histone 3, in both males and females (Figures 3A–3D). Overexpression of Drpr, which works together with Ced-6, induced ISC proliferation in young animals (Figures 3A–3D). Inhibition of *Trl*, *ced-6*, or *ci* suppressed both hyperplasia and hypertrophy during aging, which were measured by the progenitor number and size (Figures 3E, 3F, and S2C).

Since *ced-6* and *ci* are Trl target genes, we examined whether reduction of Trl expression in young progenitor cells is sufficient to decrease expression of *ced-6* and *ci*. Quantitative reverse-transcription PCR (RT-qPCR) using FACS-sorted female progenitor cells revealed that knockdown of *Trl* suppressed expression of *ced-6* but not of *ci* (Figure 3G). This indicates that *Trl* expression levels are instructive for *ced-6*, but not for *ci*, which may require additional factors such as transcription factors and epigenetic modifications. Since the causal relationship between Trl and *ced-6* is clearer, we mainly focused on *ced-6* as a Trl target in the female gut in the following investigation. We clarified that inhibition of *Trl* or *ced-6* specifically in ISCs using *Su(H)-Gal80* suppressed aging-induced ISC increase (Figures 3H and 3I) and also that inhibition of *Trl* or *ced-6* in progenitor cells inhibited hyperplasia of ISCs detected by the Delta antibody (Figures S2D and S2E). Inhibition of *Trl* or *ced-6* in progenitor cells did not induce cell death, which was detected by TUNEL, indicating that cell death does not contribute to the reduction of the progenitor cell number during aging (Figures S2F and S2G). Consistent with suppression of aging-induced ISC proliferation, *ced-6* inhibition suppressed aging-induced leakage of the blue dye from the gut (Figures S2H and S2I). We also examined whether *ced-6* or *Trl* inhibition affects organismal lifespan. Generally, an excessive proliferation of ISCs reduces lifespan; conversely, insufficient proliferation similarly leads to a shortened lifespan.^36^ Knockdown of *ced-6* or *Trl* did not affect lifespan (Figure S2J), implying that beneficial effects such as suppression of tissue dysplasia and adverse effects such as disruption of normal tissue homeostasis are canceling each other out. All together, considering that stem cell exhaustion is defined by a decline in stem cell numbers and proliferative capacity, especially in contexts of stresses,^1^^,^^9^ these data indicate that inhibition of *Trl*, *ced-6* or *ci* induces ISC exhaustion. That is, Trl and its downstream targets such as *ced-6* are necessary to suppress ISC exhaustion during aging.

**Figure 4:**
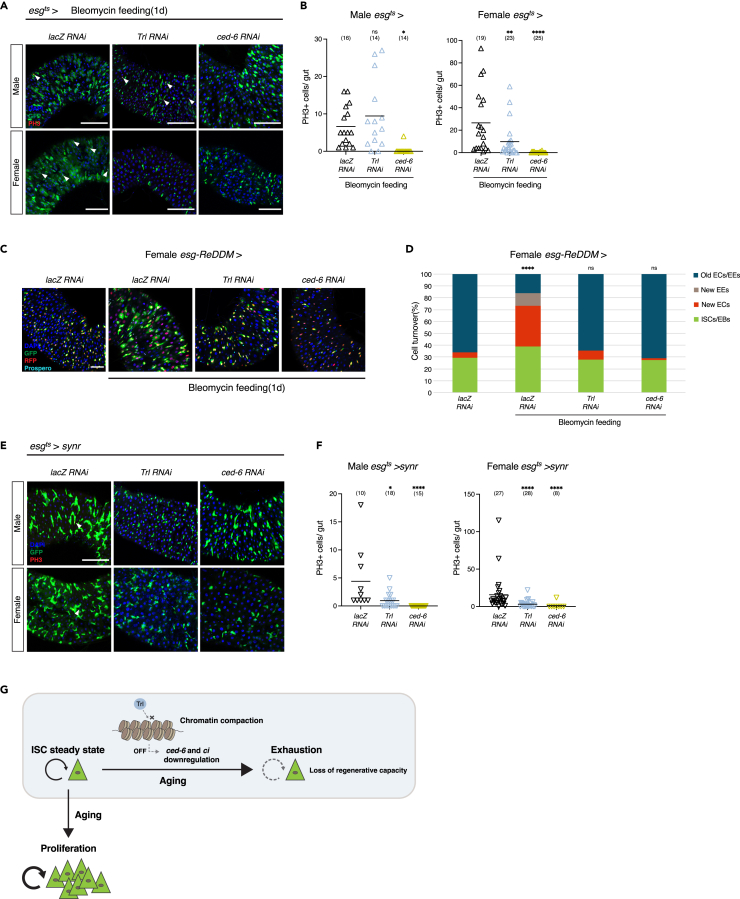
Exhaustion induced by inhibition of Trl or ced-6 makes ISCs refractory to proliferation signals induced by bleomycin or synr expression (A) Trl or ced-6 knockdown suppresses ISC proliferation induced by bleomycin feeding. Representative images of midguts showing esg-driven GFP expression (green), PH3 immunostaining (red), and DAPI (blue). Scale bars, 100 μm. Arrowheads point to PH3+ cells. (B) Quantification of PH3+ cells per midgut under the bleomycin feeding condition in male and female flies. ∗*p* = 0.0156, ∗∗*p* = 0.0055, ∗∗∗∗*p* < 0.0001; one-way ANOVA with Dunnett’s multiple comparisons test. *n* = 14–25 flies. Black lines indicate mean. (C) Trl or ced-6 knockdown suppresses an increase in differentiated cells during ISC proliferation induced by bleomycin feeding. Representative images of female midguts showing esg-ReDDM driven GFP expression (green), H2B-RFP (red, new ECs and EEs), Prospero immunostaining (cyan), and DAPI (blue). Scale bars, 50 μm. (D) Quantification of intestinal cell renewal ratio in ReDDM assay. Blue bars (old ECs and EEs), brown bars (new EEs), red bars (new ECs), green bars (ISCs and EBs). ∗∗∗∗*p* < 0.0001; Fisher’s exact test. *n* = 541–675 cells. ECs, enterocytes; EEs, enteroendocrine cells. (E) Trl or ced-6 knockdown suppresses sayonara-induced ISC proliferation. Representative images of midguts showing esg-driven GFP expression (green), PH3 immunostaining (red), and DAPI (blue). Scale bars, 100 μm. Arrowheads point to PH3+ cells. (F) Quantification of PH3+ cells per midgut when ISCs express synr in male and female flies. ∗*p* = 0.0145, ∗∗∗∗*p* < 0.0001; Kruskal-Wallis test. *n* = 10–28 flies. Black lines indicate mean. (G) Proposed model for chromatin changes during aging that skew ISC to exhaustion.

We then investigated whether Trl-mediated signaling has a general role in cellular exhaustion by exposing ISCs to stress environments that demand ISC proliferation. First, we inflicted DNA damage in enterocytes and induced ISC proliferation by feeding bleomycin.^37^ We found that knockdown of *ced-6* suppresses bleomycin-induced ISC proliferation in both males and females (Figures 4A and 4B). Knockdown of *Trl* inhibited bleomycin-induced ISC proliferation in females but not in males, indicating that there is sexual dimorphism in a role for Trl under the bleomycin-mediated stress. In the female gut, we confirmed that *Trl* or *ced-6* knockdown did not promote differentiation to either enterocytes or enteroendocrine cells but suppressed ISC proliferation by performing the ReDDM-based lineage analysis^38^ (Figures 4C and 4D). Consistent with the idea that *ced-6* is a Trl target, activation of Ced-6 by Drpr expression reversed *Trl* knockdown-mediated suppression of bleomycin-mediated progenitor cell increase (Figure S3A). Bleomycin treatment induced *Trl* downregulation and a trend of *ced-6* downregulation (Figure S3B), which might reflect the exhausted status in a similar manner to the aging situation. Next, we induced ISC proliferation genetically by overexpressing *sayonara* (*synr*) in ISCs. Synr is a BH3-only protein that we found recently.^39^ Synr activates caspases weakly and paradoxically induces ISC proliferation.^40^ We found that knockdown of *Trl* or *ced-6* suppressed ISC proliferation in both sexes when ISCs expressed Synr (Figures 4E and 4F). Synr did not affect *Trl* or *ced-6* expression (Figure S3C). These results with bleomycin and Synr suggest that the exhausted cellular status induced by inhibition of Trl or its target *ced-6* makes ISCs refractory to a variety of proliferation signals besides aging.

## Discussion

Here, using ATAC-seq and RNA-seq, we elucidated aging-induced changes of chromatin accessibility and gene expression that skew cells toward exhaustion (Figure 4G). We propose that, in parallel with aging-induced proliferation signals, chromatin structures of Trl target genes undergo compaction that suppresses gene expression, leading to insidious progress of exhaustion in ISCs during aging. Previous research has clarified many mechanisms of aging-induced ISC proliferation in *Drosophila*, but very few addressed potential exhaustion mechanisms during aging.

Regarding analyses of gene expression and chromatin accessibility in intestinal progenitor cells during aging, this is the first one to have used the progenitor cells from both females and males. Our findings that globally there is almost no correlation between gene expression and chromatin accessibility but that locally there is some correlation are consistent with the previous research with female ISCs.^22^ One intriguing aspect of our findings is that there exists strong sexual dimorphism only in changes of chromatin accessibility during aging: female, but not male, chromatin changes during aging. It will be interesting to investigate a mechanistic basis of this sexual dimorphism in the chromatin structural changes during aging.

Mechanisms of tissue stem cell exhaustion have been enigmatic. Because of that, there is no specific molecular or mechanistic marker of stem cell exhaustion. It relies on the phenotypic definition based on reduction of the stem cell number and/or of its proliferative ability that occurs in contexts of stress conditions. Exhaustion and quiescence are different in a sense that quiescent cells can respond to stress but exhausted cells cannot. We elucidated that *Trl/ced-6/ci* inhibition does not elicit an obvious phenotype in healthy young animals but that only the exhaustion phenotype becomes manifest in stressed conditions such as aging, bleomycin treatment, and Synr expression. This is consistent with the idea that ISCs with inhibition of *Trl/ced-6/ci* are not quiescent but exhausted, unable to cope with demands by stresses.

The observation that *ced-6/ci* inhibition suppresses ISC proliferation while *ced-6/ci* expression decreases during aging might be confusing at a glance. But, we propose that downregulation of Trl target *ced-6* or *ci* skews ISCs toward exhaustion during aging but that the signals that lead cells toward proliferation are too strong and mask the exhaustion aspect. This study uncovers previously unsuspected insidious events that occur in chromatin during aging.

Although how Ci regulates ISC proliferative capacity has been elucidated,^32^ it remains unclear how Ced-6 and Drpr, which regulate cellular engulfment,^29^^,^^30^^,^^31^ mediates ISC homeostasis. Future research needs to address this mechanism.

At present, we do not know whether aging-induced changes of chromatin structures and transcription in Trl target genes are adaptive or not for the organism. We speculate two possibilities: the chromatin and gene expression changes that skew cells toward exhaustion may be a counteractive mechanism for aging-induced dysplasia or just a byproduct of a non-adaptive deteriorating event that occurs during aging.

### Limitations of the study

Although we propose that two opposing signals, which induce proliferation and exhaustion, occur in ISCs during aging, whether the two signaling events occur in the same cells or in different cells is not clarified. Future studies based on single cell-based approaches will be able to clarify this aspect. In this study, cellular exhaustion is defined phenotypically based on a decline in stem cell numbers and proliferative capacity in contexts of stresses and not based on a molecular marker. Finding a molecular marker of exhaustion will further accelerate mechanistic understanding of cellular exhaustion.

## Resource availability

### Lead contact

Further information and requests for resources and reagents should be directed to and will be fulfilled by the lead contact, Sa Kan Yoo (sakan.yoo@riken.jp).

### Materials availability

This study did not generate new unique reagents.

### Data and code availability

• Raw ATAC-seq and RNA-seq data have been deposited at SRA. Accession numbers are listed in the key resources table.
• This paper does not report original code.
• Any additional information required to reanalyze the data reported in this paper is available from the lead contact upon request.

## Acknowledgments

We thank Iswar Hariharan, Donald Fox, Norbert Perrimon, Yuichiro Nakajima, Fumiaki Obata, and TRiP at Harvard Medical School; the Bloomington Stock Center; and the VDRC stock center for fly stocks. We thank BDR’s DNA sequencing technical support team for the DNA library preparation. We thank the Yoo lab members, Fumiaki Obata, Asami Oji, and Ichiro Hiratani for helpful comments on the manuscript. This work was supported by RIKEN Junior Research Associate Program and JST SPRING (JPMJSP2138) to S.T.-N. and AMED-PRIME (17939907), 10.13039/501100001691JSPS KAKENHI (JP16H06220), 10.13039/501100001691JSPS KAKENHI (JP22H02807), and JST FOREST (JPMJFR216F) to S.K.Y.

## Author contributions

S.T.-N.: conceptualization, data curation, formal analysis, investigation, methodology, and writing – review and editing. S.S.: investigation, methodology, and writing – review and editing. S.K.Y.: conceptualization, supervision, funding acquisition, investigation, methodology, writing – original draft, project administration, and writing – review and editing.

## Declaration of interests

The authors declare no competing interests.

## STAR★Methods

### Key resources table

**Table:** 

REAGENT or RESOURCE	SOURCE	IDENTIFIER
**Antibodies**		

Rabbit-anti-phospho-H3	Millipore	Cat #06-570, RRID:AB_310177
Mouse-anti-Delta	DSHB	Cat #c594.9B, RRID:AB_528194
Mouse-anti-Prospero	DSHB	Cat #MR1A, RRID:AB_528440
Alexa Fluor 488 goat anti-rabbit IgG	Invitrogen	Cat #A11008, RRID:AB_143165
Alexa Fluor 568 goat anti-mouse IgG	Invitrogen	Cat #A11004, RRID:AB_2534072

**Chemicals, peptides, and recombinant proteins**		

Bleomycin sulfate	TCI	Cat #B3972
Brilliant Blue FCF	Wako	Cat #027-12842
PrimeScript RT Master Mix	TaKaRa	Cat #RR036A
FastStart Essential DNA Green Master	Roche	Cat #06402712001

**Critical commercial assays**		

Maxwell RSC simplyRNA Tissue Kit	Promega	Cat #AS1340
Arcturus PicoPure™ RNA Isolation Kit	Applied Biosystems	Cat #KIT0204
Arcturus RiboAmp HS PLUS RNA Amplification Kit	Applied Biosystems	Cat #KIT0525
ApopTag Red *in situ* Apoptosis Detection Kit	Millipore	Cat #S7165

**Deposited data**		

Raw data of ATAC-seq	This paper	SRA:PRJNA1137551 https://www.ncbi.nlm.nih.gov/bioproject/1137551
Raw data of RNA-seq	This paper	SRA: PRJNA1138800 https://www.ncbi.nlm.nih.gov/bioproject/1138800

**Experimental models: Organisms/strains**		

*D. melanogaster: esg-GFP*	Donald Fox(Duke University, USA)	P01986
*D. melanogaster: Esg-Gal4, UAS-GFP, tubgal80ts*	Norbert Perrimon(Harvard University, USA)	N/A
*D. melanogaster: Esg-Gal4, UAS-GFP; tubgal80ts, Su(H)-gal80*	Norbert Perrimon(Harvard University, USA)	N/A
*D. melanogaster: Esg-Gal4, UAS-GFP, tubgal80ts; UAS-synr*	Sulekh et al.^40^	N/A
*D. melanogaster: Esg-Gal4, UAS-mCD8-GFP, UAS-H2B-RFP, tubgal80ts*	Yuichiro Nakajima(The University of Tokyo, Japan)	N/A
*D. melanogaster: UAS-ced-6 RNAi*	Vienna *Drosophila* Resource Center	VDRC:108101
*D. melanogaster: UAS-ced-6 RNAi*	Vienna *Drosophila* Resource Center	VDRC:16313
*D. melanogaster: UAS-Trl RNAi*	Vienna *Drosophila* Resource Center	VDRC:41095
*D. melanogaster: UAS-ci RNAi*	Bloomington *Drosophila* Stock Center	BDSC:31236
*D. melanogaster: UAS-lacZ RNAi*	Fumiaki Obata(RIKEN, Japan)	N/A
*D. melanogaster: UAS-drpr*	Bloomington *Drosophila* Stock Center	BDSC:67035
*D. melanogaster: w*^*1118*^	Erina Kuranaga(Kyoto University, Japan)	N/A
*D. melanogaster:OregonR*	Iswar Hariharan(University of California Berkeley, USA)	N/A
*D. melanogaster:w*^*OregonR*^*(w*^*1118OregonR*^*)*	Sasaki et al.^16^	N/A

**Oligonucleotides**		

Primer for *RpL32* (forward)	CCAGCATACAGGCCCAAGATCGTG	N/A
Primer for *RpL32* (reverse)	TCTTGAATCCGGTGGGCAGCATG	N/A
Primer for *ced-6* (forward)	GCAACGGGGATGCCAAAAG	N/A
Primer for *ced-6* (reverse)	CGCTCAGGTTACCAAAGAACTTG	N/A
Primer for *Trl* (forward)	TTTCCCGCCCACAAGATAG	N/A
Primer for *Trl* (reverse)	CCAGATCGTTCGCATTGACG	N/A
Primer for *ci* (forward)	GATTTTCGCCAAACTCTTTAGCC	N/A
Primer for *ci* (reverse)	ACATGGGATTAAGGGCGGTAG	N/A

**Software and algorithms**		

Prism v10.2	Graphpad	N/A
ImageJ v2.3.0	Wayne Rasband, NIH	https://imagej.nih.gov/ij
CLC Genomics Workbench software v20.0.4	Filgen	N/A
MACS2 v2.1.0 v2.2.7.1	Zhang et al.^41^	https://pypi.org/project/MACS2/
Homer v4.10	Chris Benner, University of California San Diego	http://homer.ucsd.edu/homer/motif/
iDEP v0.85	Ge et al.^23^	http://ge-lab.org/idep/

### Experimental model and study participant details

Flies were maintained as previously described.^42^ The fly food was composed of 0.8% agar, 10% glucose, 4.5% corn flour, 3.72% dry yeast, 0.4% propionic acid and 0.3% butyl p-hydroxybenzoate. For the temperature-shift experiments, flies were raised at 18°C and virgin females and males were collected and moved to 30°C 4-7 days after eclosion. Genes were induced at 30°C for 5 days (young) and for 20 days (old). In the bleomycin feeding, approximately 10 day-old flies were fed with 25 μg/ml Bleomycin (TCI, B3972) dissolved in 5% sucrose agar food for 1 day at 25°C after 5 day-gene induction at 30°C. For the ReDDM assay, flies were raised at 18°C, moved to 30°C, incubated for 6 days, fed with Bleomycin for 1 day and dissected 3 days after the Bleomycin treatment.

### Method details

#### ATAC-seq analysis

To perform ATAC-seq, intestinal progenitor cells were isolated by Fluorescent Activated Cell Sorting (FACS). Male *esg-GFP* flies were crossed to female OreR and the progeny were placed at 25°C until 7d-old or 8w-old. Midguts from female and male *esg-GFP* expressing fly were dissected in 1x PBS and dissociated in 1 mg/ml Elastase solution (Wako) for 1h at room temperature. The dissociated cells were re-suspended in 1xPBS after centrifuge, filtered through a 40μm filter, and kept on ice until sorting. Cell sorting was performed using a SH800S cell sorter (SONY). Sorted cells were re-suspended in 50% FBS, 10% DMSO and 40% Drosophila Schneider medium after centrifuge and stored at -80°C. Two replicates were prepared in each sample. 50,000 -100,000 cells were shipped to Active Motif. Sample preparation and sequencing analysis for ATAC-seq were performed by Active Motif. Sequencing was performed by Illumina sequencing using NextSeq 500. Peaks were called using MACS2(v2.1.0).

#### RNA-sequencing

10,000-50,000 intestinal progenitor cells were collected by FACS as described above. Total RNA was isolated using the Arcturus PicoPure RNA Isolation Kit (Applied Biosystems). To obtain enough RNA amount for sequencing, isolated RNA was amplified using the Arcturus RiboAmp HS PLUS RNA Amplification Kit (Applied Biosystems). The amplification process was completed in the 1st round to suppress artificial RNA amplification. Library preparation was performed using KAPA mRNA HyperPrep Kit (Roche). The prepared libraries were sequenced by the HiSeq X (GENEWIZ outsource). Read mapping and counting were carried out with CLC Genomics Workbench software (v20.0.4) (Filgen).

#### Bioinformatics analysis

Active Motif performed bioinformatics analysis of ATAC-seq analysis including differential analysis and PCA. Differential expression analysis, PCA for RNA-seq analysis and TF binding motif enrichment analysis on differentially expressed gene promoters were performed using the iDEP web application for RNA-seq data analysis (v0.85).^23^ TF binding motif enrichment analysis on differentially accessible promoter peaks was calculated with Homer (v4.10). To obtain promoter peak data, the following procedures were performed; peak calling using MACS2(v2.2.7.1) and peak merging from each replicant into unit peak data using Homer utility mergePeaks.

#### Immunofluorescence and imaging

Whole midguts were dissected in PBS and fixed in a fixative solution (4% paraformaldehyde in PBS) for 1h. The samples were washed in PBS with 0.1% Triton X-100 for 10 min at RT. Primary antibody was diluted in PBS with 0.1% Triton X-100 and 10% normal goat serum and samples were incubated overnight at 4°C. After washing, the samples were incubated with secondary antibody and DAPI for 4h at RT. The following reagents were used at indicated dilution: rabbit-anti-phospho-H3(1:1,000, Millipore, 06-570), mouse-anti-Delta (1:20, DSHB, c594.9B), mouse-anti-Prospero(1:20, DSHB, MR1A), Alexa Fluor 488 goat anti-rabbit IgG (1:500, Invitrogen, A11008), Alexa Fluor 568 goat anti-mouse IgG (1:500, Invitrogen, A11004), and DAPI (1:500, Sigma, D9542). Fluorescence images were acquired with a confocal microscope (Ziess LSM900).

#### Quantitative PCR with reverse transcription

Expression level of *ced-6* and *ci* in *Trl* knockdown intestinal progenitor cells were measured by RT-qPCR. 60,000-45,000 progenitor cells were collected from young *esg*^*ts*^>*lacZ RNAi* or *Trl RNAi* by FACS. Total RNA was isolated using the Arcturus PicoPure RNA Isolation Kit (Thermo Fisher Scientific). 3 replicates were used for each condition. For measuring the expression level of *Trl* and *ced-6* under bleomycin-fed or *synr* overexpression conditions, midguts of 1d-bleomycin-fed or *synr*-expressing *esg*^*ts*^>*lacZ RNAi* flies were dissected. Total RNA was extracted from 5 midguts per sample by using the Maxwell RSC simplyRNA Tissue Kit (Promega). 5 replicates were used for each condition. Expression levels of *ced-6* were measured by RT-qPCR of mRNA from L3 whole body (*act>UAS RNAi*). Total RNA was extracted from the whole body of wandering L3 larvae by using the Maxwell RSC simplyRNA Tissue Kit (Promega). 250ng of extracted RNA was reverse transcribed using PrimeScript RT Master Mix (RR036A, TaKaRa). Real-time PCR was performed using FastStart Essential DNA Green Master (Roche) with Light Cycler 96 (Roche). 7 replicates were used for each condition. Transcript levels were normalized with RpL32 in the same samples.

#### Terminal deoxynucleotidyl transferase dUTP nick end labeling (TUNEL) assay

The TUNEL assay was performed using the ApopTag Red *in situ* apoptosis detection kit (Millipore) as described previously.^39^ Adult midguts of 5d and 20d-old flies were dissected in 1×PBS and fixed for 30 min in 1×PBS with 4% paraformaldehyde at room temperature (RT). After fixation, samples were washed with PBS 0.1% Triton-X100 and incubated in equilibration buffer (Apop Tag kit; Millipore) for 10 s. Then, samples were incubated in reaction buffer (TdT enzyme; ratio 7:3; Apop Tag kit) at 37°C for 1 h. The TdT reaction mix was replaced with stop buffer (diluted 1:34 in dH2O; Apop Tag kit) and incubated for 10 min at RT. Samples were then washed with PBS 0.1% Triton-X100 3 times and incubated with antidigoxigenin antibody solution (diluted 31:34 in blocking solution; ApopTag kit) overnight at 4°C. Samples were then washed with PBS 0.1% Triton-X100 3 times again and mounted on the slide glass.

#### Lifespan analysis

For lifespan measurement, virgin female *Trl* or *ced-6* knockdown flies were collected for 4-7 days after eclosion and placed at 18°C. After that, flies were placed at 29°C. Food was replaced every 2-3 days throughout the life.

#### Smurf assay

Blue dye food was prepared using SY food (5% sugar and 10% yeast) with Brilliant Blue FCF(Wako) at a concentration of 2.5%(wt/vol). Female *ced-6* knockdown flies were collected for 4-7 days after eclosion and placed at 18°C. After that, flies were transferred to blue dye food and placed at 29°C. Blue dye food was replaced every 2-3 days. Counting the number of smurf flies that show dye leakage in whole abdomen was conducted on day 5 and 20.

### Quantification and statistical analysis

Quantification of the cell number and size was performed by ImageJ software. Cell density was calculated by dividing the number of cells by tissue area (approximately 25000 μm^2^). Midgut pictures for quantification were taken in R4a-b region unless otherwise described.

GraphPad Prism 10.2 and R were used for statistical analysis. For single comparisons, data sets were analyzed by two-sided unpaired t-test or Mann-Whitney test. Multiple comparisons were analyzed by one-way ANOVA or Kruskal-Wallis tests. Ratio comparisons were analyzed by Fisher’s exact test. Multiple testing was performed with Benjamini-Hochberg procedure. For survival analyses, log-rank (Mantel-Cox) test was performed. Statistical tests and significance used are indicated in the figure legends. Sample sizes were determined empirically based on the observed effects.
