# Supplementary material for: Insidious chromatin change with a propensity to exhaust intestinal stem cells during aging
Source: iScience. 2024 Sep 9;27(9):110793. doi: 10.1016/j.isci.2024.110793 (PMC11452737; doi:10.1016/j.isci.2024.110793)
Supplement: Document S1. Figures S1–S3 [file mmc1.pdf]

iScience, Volume *27*

Supplemental information

Insidious chromatin change with a propensity to exhaust intestinal stem cells during aging

Saki Tomita-Naito, Shivakshi Sulekh, and Sa Kan Yoo

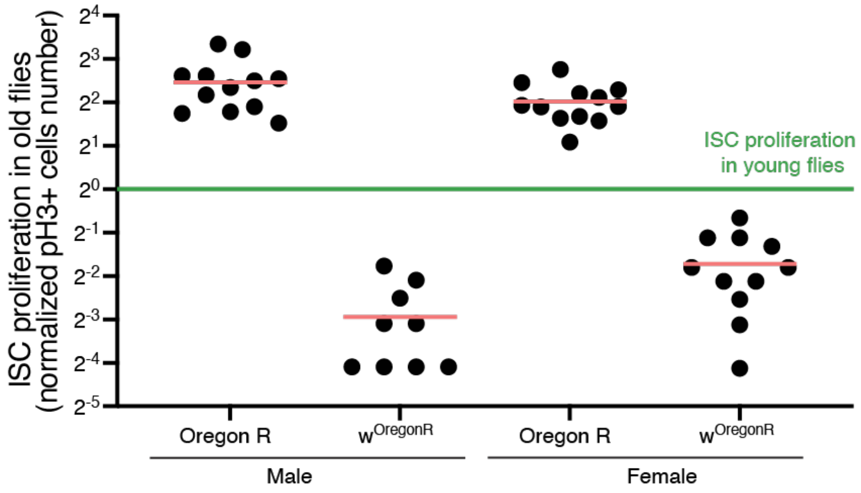

Figure S1. Data of the white mutant, Related to Figure 1

The *white* mutant demonstrates reduction of ISC proliferation during aging, which contrasts with the increase of ISC proliferation during aging in OregonR. n = 9-12 flies. Pink lines indicates mean.

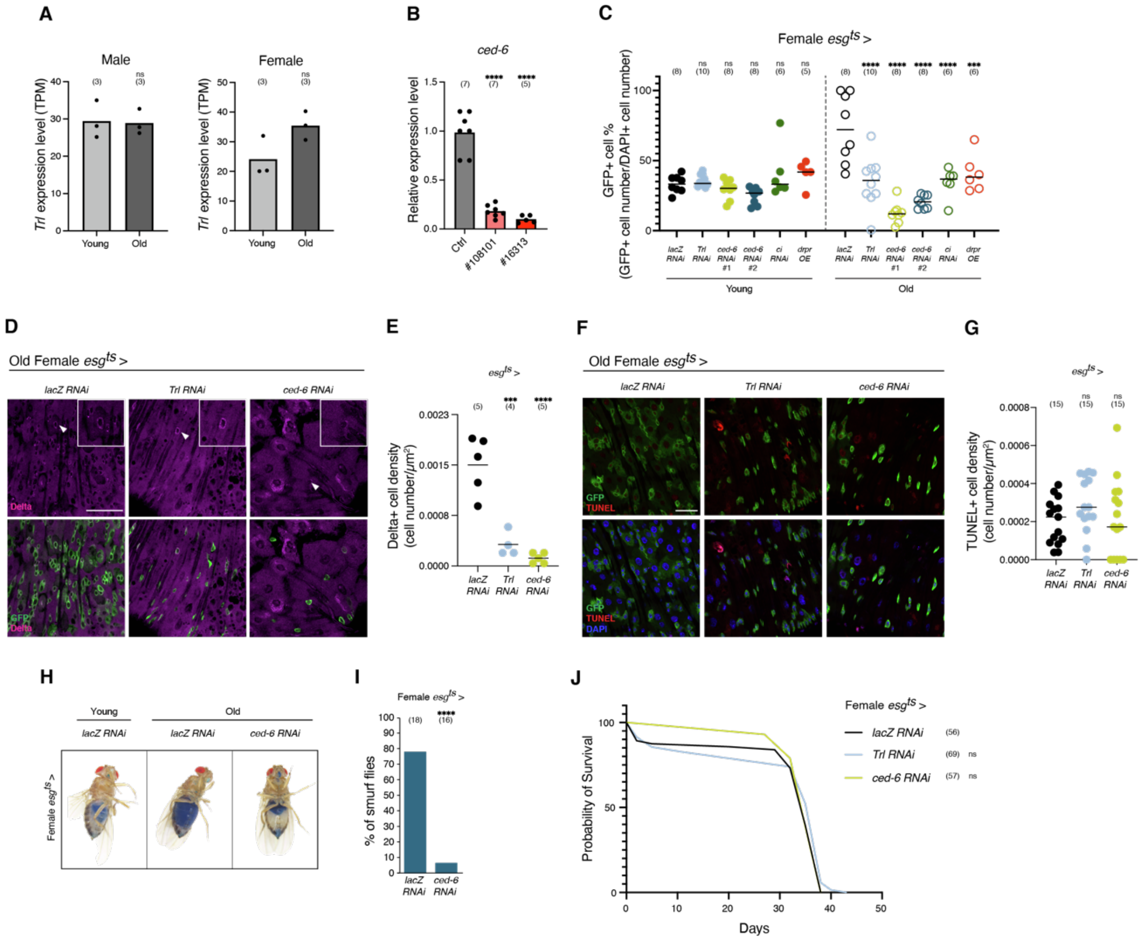

Figure S2. Trl, ced-6 and ci are involved in ISC exhaustion, Related to Figures 2-3

A. *Trl* expression levels from RNA-seq data in male and female intestinal progenitors. Two-tailed unpaired t-test. n = 3 replicates. Bars indicate mean.

B. Knockdown efficiency of *ced-6* RNAis was validated by RT-qPCR with mRNA from *act>RNAi* L3 larvae. ****P<0.0001; one-way ANOVA with Dunnett's multiple comparisons test. n = 5-7 replicates. Bars indicate mean.

C. Quantification of percentage of GFP+ cells per DAPI+ cells in imaged areas in female midguts (young and old). ***P<0.001, ****P<0.0001; one-way ANOVA with Dunnett's multiple comparisons test. n = 5-10 flies. Black lines indicate mean.

D. Representative images of midguts showing *esg*-driven GFP expression (green) and Delta immunostaining (red). Arrowheads indicate ISCs. Scale bars, 50µm.

E. Quantification of Delta+ cell density. ***P<0.001, ****P<0.0001; one-way ANOVA with Dunnett's multiple comparisons test. n = 4-5 flies. Black lines indicate mean.
F. Representative images of midguts showing *esg*-driven GFP expression (green), TUNEL staining (red) and DAPI (blue). Scale bars, 30μm.
G. Quantification of TUNEL+ cell density. one-way ANOVA with Dunnett's multiple comparisons test. n = 15 flies. Black lines indicate median.
H. Representative images of smurf assay in *ced-6* knockdown flies (5 or 20 day-old).
I. Quantification of flies showing the smurf phenotype at the old age (20 day-old). ****P<0.0001; Fisher's exact test. n = 16-18.
J. Survival curve of *Trl* or *ced-6* knockdown flies. Black line (*lacZ* KD), blue line (*Trl* KD), yellow line (*ced-6* KD). Log-rank test. n = 56-69 flies.

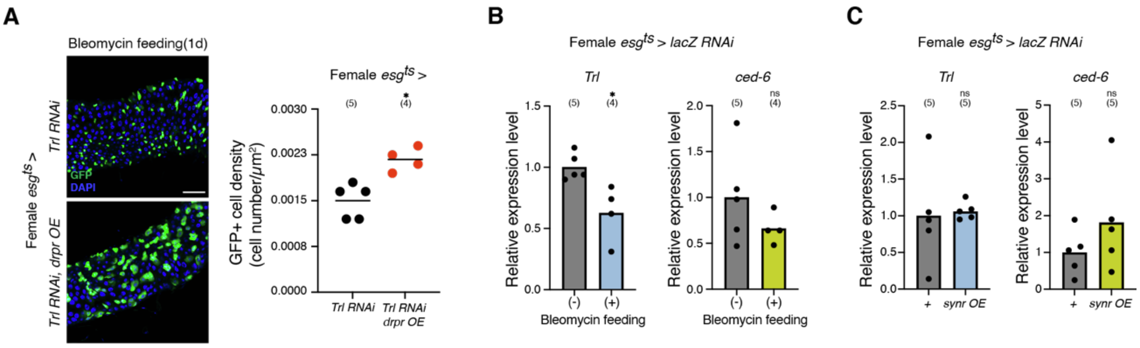

Figure S3. Roles for Trl and ced-6 in responses to bleomycin and sayonara, Related to Figure 4

A. *drpr* overexpression rescued proliferative defect of *Trl* knockdown flies under the bleomycin-fed condition. Representative images of the midgut showing *esg*-driven GFP expression (green) and DAPI (blue). Scale bars, 50µm. The graph shows the quantification of GFP+ cell density. *P=0.0159; Mann-Whitney test. n = 4-5 flies. Black lines indicate mean.

B. RT-qPCR of *Trl* and *ced-6* mRNA in midguts isolated from bleomycin-fed control flies (*esg*^{ts}>*lacZ* RNAi). 5 midguts/sample. *P=0.0140; two-tailed unpaired t-test. n = 4-5 replicates. Black lines indicate mean.

C. RT-qPCR of *Trl* and *ced-6* mRNA in midguts isolated from *synr*-overexpressing flies (*esg*^{ts}>*lacZ* RNAi and *UAS-synr*). 5 midguts/sample. two-tailed unpaired t-test. n = 5 replicates. Black lines indicate mean.
